# Fludarabine combined with radiotherapy in patients with locally advanced NSCLC lung carcinoma: a phase I study

**DOI:** 10.1007/s00432-012-1185-3

**Published:** 2012-03-10

**Authors:** Mirko Nitsche, Hans Christiansen, Katinka Lederer, Frank Griesinger, Heinz Schmidberger, Olivier Pradier

**Affiliations:** 1 Center of Radiation-Oncology, Gröpelinger Heerstr. 406-408, 28239 Bremen, Germany; 2 Department of Radiation-Oncology, Georg-August University of Göttingen, Robert Koch Str. 40, 37075 Göttingen, Germany; 3 Department of Radiotherapy, Medical School Hanover, Carl-Neuberg-Str. 1, 30625 Hannover, Germany; 4 Department of Radiation-Oncology, Sykehuset Innlandet HF, 2816 Gjøvik, Norway; 5 Department of Hematology and Oncology, Georg-August University of Göttingen, Robert Koch Str. 40, 37075 Göttingen, Germany; 6 Department of Hematology and Oncology, Klinikum Oldenburg, Dr.-Eden-Str. 10, 26133 Oldenburg, Germany; 7 Department of Radiation-Oncology, Johannes Gutenberg University of Mainz, Langenbeckstr. 1, 55131 Mainz, Germany; 8 Département de Cancérologie, Centre Hospitalier Universitaire de Brest, 2, avenue Foch, 29609 Brest cedex, France

**Keywords:** Fludarabine, NSCLC, Nucleoside analogue, Concurrent fludarabine and radiotherapy, Radiotherapy, phase I study, Radiochemotherapy in stage III NSCLC, locally advanced inoperable NSCLC

## Abstract

**Background and purpose:**

Fludarabine is an adenine nucleoside analogue that has significant activity in hematological malignancies and has shown promising activity in combination with radiation in preclinical solid tumor models. We designed a phase I trial exploring concurrent fludarabine and radiotherapy in patients with advanced non-small cell lung cancer (NSCLC) to determine the maximum tolerated dose (MTD) of fludarabine given with concurrent irradiation.

**Materials and methods:**

Thirteen patients with stage IIIB NSCLC received thoracic irradiation of 60 Gy. Fludarabine was administered during the 5th and 6th week of radiotherapy. Doses started at 10 mg/m^2^ per day and increased by steps of 3 mg/m^2^ per day.

**Results:**

At a daily dose of 16 mg/m^2^, one out of six patients developed a grade 4 leukopenia, and one a grad 3 pneumonitis. Further grade III toxicity was not observed. The dose of 13 mg/m^2^ was identified as the MTD. All patients developed a fludarabine dose-dependent lymphocytopenia.

**Conclusion:**

Fludarabine can be safely administered concurrently with radiation at a daily dose of 13 mg/m^2^ during the final 2 weeks of radiotherapy. Further prospective clinical studies are required to establish the potential role of concurrent fludarabine and radiotherapy in the treatment of locally advanced inoperable NSCLC.

## Introduction

Despite the advances in multimodal therapy, locally advanced stage IIIA and IIIB non-small cell lung cancer (NSCLC) remains a disease with a poor overall prognosis, and the optimal treatment still remains to be investigated (Buccheri and Ferrigno 1996; Spira and Ettinger 2004). With conventional radiotherapy (RT) alone, both local and distant failure rates are high, and the expected median survival is generally between 9 and 12 months (Perez et al. 1987). To improve local control rates and survival, surgery following induction chemotherapy or chemoradiotherapy regimen can be attempted (Ginsberg 1995; Seung and Ross 2009; Edelman et al. 2008). However, the following seems to be the most promising therapy for the majority of patients with locally advanced inoperable stage IIIA and IIIB NSCLC: Several trials evaluated radiotherapy with concurrent cisplatin-based chemotherapy and revealed better survival rates than RT alone, even in comparison with sequential therapy (Le Chevalier et al. 1991; Schaake-Koning et al. 1992; Sause et al. 1995; Furuse et al. 1999; Curran 2002, O’Rourke et al. 2010). However, the concurrent approach appears to increase the rate of adverse events, mainly esophagitis. Providing support for these results, two meta-analyses showed a significant decrease in the relative risk of death at 1 and 3 years and also a 24 % reduction in the risk of death at 1 year and a 30 % reduction at 2 years for radiotherapy with concurrent cisplatin-based chemotherapy in comparison with radiotherapy alone (Pritchard and Anthony 1996; Marino et al. 1995). Multiple cisplatin-based drug combinations for concurrent chemoradiotherapy have been investigated to improve those results. Mainly docetaxel, etoposide, topotecan, vinorelbine, paclitaxel, irinotecan, navelbine and gemcitabine are applied, with different outcomes in local control and overall toxicity (Nakamura et al. 2008; Seung and Ross 2009; Naito et al. 2008; Kelly et al. 2008; Kosmidis et al. 2007; Choong et al. 2005). Gemcitabine, for example, has shown excellent activity not only in sequential chemoradiotherapy but also in concurrent chemoradiotherapy for NSCLC. Results from phase I/II trials support its efficacy, but indicate also significant toxicity (Abacioglu et al. 2005; Blanco et al. 2008; Curran 2002). Fludarabine does imply nearly the same mechanisms of action as gemcitabine, inhibiting various enzymes involved in DNA replication, and is therefore investigated in our study for effectiveness and toxicity.

*Fludarabine*-*phosphate* (*fludarabine*) is a single phosphorylated and fluoridated adenine nucleoside derivative (9-β-d-arabinofuranosyl-2-fluoroadenine-5′-monophosphate), which is established in the treatment of chronic lymphatic leukemia (Johnson et al. 1996; Keating et al. 1989). Fludarabine-phosphate is a prodrug, which is rapidly dephosphorylated in vivo to 2-F-ara-A. The dephosphorylated drug is actively transported into the cell, whereupon it is rephosphorylated to fludarabine-triphosphate (2-F-ara-ATP) (Brockman et al. 1980; Plunkett et al. 1980). 2-F-ara-ATP, the active form, inhibits enzymes that are involved in DNA synthesis and DNA repair like DNA polymerase alpha and epsilon DNA primase and ligase and the ribonucleotide reductase (Plunkett et al. 1990; Plunkett et al. 1993). The drug is also incorporated in the DNA and induces a termination of the chain elongation (Huang et al. 1990). Inhibition of DNA repair is a well-known mechanism of radiosensitization, and some investigations have demonstrated that drugs like Ara-A, which inhibits the DNA rejoining, are also able to enhance the cytotoxic effect of radiation (Dahlberg and Little 1992; Malaise et al. 1989). It was also demonstrated that fludarabine inhibits the repair of radiation-induced damage of chromosomes in human peripheral blood cells (Jayanth and Hittelman 1991). First studies with fludarabine–p in vitro were performed in 31 different cell types of cancer. Continuous exposure at high concentrations (1.0 μg/ml) resulted in a notable cytotoxic activity against acute leukemia and non-Hodgkin’s lymphomas, whereas no effect was seen against the majority of cell lines from solid tumors (Lathan et al. 1988). The antitumor activity of fludarabine has been studied against all major tumor types in clinical phase II trials. However, the results were generally disappointing, only in head and neck cancer and breast cancer, a small proportion of patients had objective remissions (Weiss et al. 1987; Mittelmann et al. 1988). Pointing out the potential effect of fludarabine as a radiosensitizer, different animal experiments were performed. It has been shown that fludarabine in vivo is a potent enhancer of radiation effectiveness in several mouse tumor models after single and fractionated irradiation (Gregoire et al. 1994a, b, c, Kim et al. 1986). Fludarabine has also been reported to increase radiation-induced clonogenic cell death in several mouse sarcoma cell lines in vitro (Laurent et al. 1998). This effect was beyond that expected by additivity (van Putten et al. 2003). Further on, there is in vitro data about radiosensitizing in several squamous carcinoma cell lines (Nitsche et al. 2008; Gregoire et al. 2002). However, the effectiveness of fludarabine in combination with radiation on solid tumor cells still in vivo has still to be proven. As a recent phase I study demonstrated that fludarabine can be safely administered concurrently with radiation, we actuated our phase I study (Jeremic et al. 1993). The rationale for the application of fludarabine as a radiosensitizer in our study was as follows: Fludarabine was administered during week 5 and 6 of radiotherapy in order to introduce a second mode of action, since the remaining or repopulating tumor cells after 4 weeks of radiotherapy would have a higher proliferative activity and therefore increased susceptibility against fludarabine. Further, the potential neurotoxicity of fludarabine in conjunction with irradiation, which was observed in animal studies, led us to apply the drug at a time when the spinal cord was spared in the irradiated volume. The primary objective of our study was to determine the MTD for a daily schedule of fludarabine during 2 weeks of the thoracic radiotherapy for patients with irresectable stage III NSCLC.

## Patients and methods

Eligibility was as follows: age ≥18 years; histologically confirmed advanced NSCLC classified as inoperable stage IIIA or stage IIIB by the UICC System, a Karnofsky performance score (KPS) of ≥70 %, and no previous therapy. Patients were excluded if they had postoperative thoracic recurrence or a history of any prior or concurrent cancer (except that of the skin) within the past 5 years. Patients with malignant pleural effusion were also excluded.

The pretreatment evaluation included medical history, physical examination, complete blood count, biochemical screening tests, pulmonary function tests, posteroanterior and lateral chest radiography, and computed tomography (CT) of the thorax and upper abdomen. Brain CT scanning was performed only if patients showed clinical symptoms of CNS involvement.

### Radiotherapy

RT was administered with 6 or 20-MV photons using linear accelerators. For dosimetry, computed tomography scans were obtained for all patients. The planning target volume encompassed the primary tumor plus involved and/or elective lymph nodes with a minimum margin of 2 cm. The initial planning target volume was treated with a minimum dose of 50 Gy. Afterward the target volume was reduced to the plain macroscopic tumor volume detectable on CT scan and irradiated to a total dose of 60 Gy. Doses were specified at middepth at central axis for parallel-opposed fields or at the intersection of central axes for other techniques, as specified in the ICRU 50 report. Normal tissue tolerance criteria for the heart, spinal cord, involved and uninvolved lung were mandated as follows. For the spinal cord, the maximum dose was limited to 45 Gy. The dose to the entire heart was limited to 35 Gy.

Since the lung tolerance to irradiation is depending on the percentage of lung volume involved in radiation fields, the percentage of the total lung receiving >20 Gy was limited at 30–35 % of the total lung volume. The patients were treated with a daily fraction of 2.0 Gy.

### Fludarabine administration

Fludarabine (Fludara, Schering AG) was reconstituted in saline and given as an intravenous infusion over 30 min. Fludarabine was administered i.v. daily 3–4 h before each fraction of radiotherapy, for the last 10 fractions of the treatment. The starting dose was 10 mg/m^2^ per day and increased by steps of 3 mg/m^2^ per day for the first 3 steps and by steps of 2 mg/m^2^ per day up to the MTD. It was, however, decided beforehand to stop the trial at a daily dose of 20 mg/m^2^ (total dose of 200 mg/m^2^) to avoid possible neurological complications.

The treatment scheme is shown in Table 1.

**Table 1:** Treatment schema

Week	1	2	3	4	5	6 [Boost]
Fludarabine	–	–	–	–	| | | | |	| | | | |
Radiotherapy	| | | | |	| | | | |	| | | | |	| | | | |	| | | | |	| | | | |

Fludarabine 10, 13, or 16 mg/m^2^ i.v. 3–4 h before radiotherapy, day 1–5, week 5–6, radiotherapy 2 Gy/day, 5 days/week to 60 Gy

### Monitoring of side effects

Patients were monitored weekly for the first 4 weeks of the treatment, and three times a week for the last 2 weeks, when fludarabine was administered. Blood counts including differential blood stains and the determination of the number of CD4 and CD8 lymphocytes by fluorescent cytometry were obtained weekly during radiotherapy and twice a week during combined treatment with fludarabine and radiation. After treatment, the monitoring of blood counts was continued until recovery to normal values could be observed.

CD4 and CD8 lymphocytes were evaluated on a Beckmann-Coulter EPICS XL MCL FACS-Scan. 100 μl heparinized peripheral blood and 10 μl of antibodies (Coulter Cyto-STAT-tetraCHROME™: CD45 FITC, CD4-RD1, CD8-ECD, CD3-PC5) were incubated in TQ-Prep Workstation automated lysing device for 10 min, and erythrocytes were lysed by Immuno-Prep lysing reagent. Afterward, 100 μl of flow-count beads (Clow-Count™ Flourosperes) were added. Relative and absolute CD4 and CD8 counts were measured on the EPICS using an automated gating programme based on a CD45 gating algorithm (tetraONE SYSTEM Software).

The MTD was defined as the highest dose of fludarabine that could be safely administered to a patient in combination with radiotherapy, producing tolerable, manageable, and reversible toxicity. The assessment of MTD was based on acute toxicity according to the criteria of the Radiation Therapy Oncology Group (RTOG) and the European Organization for the Research and Treatment of Cancer (EORTC) scores (Cox et al. 1995).

The MTD was defined as the dose inducing grade 4 hematological (neutropenia or thrombocytopenia), or grade 3 skin, mucosal or lung toxicity in at least 1 of 3 patients or at least 2 of 6 patients per level. When only one out of three patients at a dose level presented with grade 3 (non-hematological) or 4 (hematological) toxicity, an additional three patients were included at the same dose level to confirm the MTD.

### Response evaluation and follow-up

Response evaluation was performed at 6 weeks after completion of the treatment by physical examination and CT scans of the thorax. Thereafter, patients were scheduled for follow-up examinations every 2 months for the first 2 years, and every 6 months after 2 years. A medical history, physical examination, complete blood count, biochemical tests, and chest radiography were performed at each visit. At the time of any progression, restaging was performed with CT scans of the thorax in each patient. Further examinations such as bone scans, brain imaging, or abdominal ultrasound were performed depending on the clinical complaints of the patients. Patients with metastatic disease were offered palliative chemotherapy.

## Results

### Patient characteristics

Between March 1999 and July 2003, 13 patients were treated within this study at the University of Göttingen. Patient characteristics are given in Table 2. There were 12 men and one women. Three patients had stage IIIA, and 10 stage IIIB. All patients had squamous cell carcinoma. All patients had a Karnofsky performance status of >80 %, and only 3 patients experienced a weight loss of ≥5 %. Of the 13 patients, 4 had clinically N2 positive disease and 7 had N3 positive disease. The lymph node status was defined with computer tomography and/or mediastinoscopy.

**Table 2:** Toxicity

Case no.	Fludarabine dose level (mg/m^2^/day)	TNM-staging^a^	Grade of local toxicity [RTOG/EORTC criteria]			Grade of hematological toxicity [nadir] [WHO criteria]			
			Skin	Esophagus	Lung	Anemia	Neutropenia	Lymphopenia	Thrombopenia
1	10	T2N2	0	1	1	0	0	4	0
2	10	T4N0	1	1	1	0	0	4	0
3	10	T4N3	1	1	1	2	1	4	0
4	13	T2N3	1	1	0	0	0	4	0
5	13	T4N2	0	2	0	0	1	4	0
6	13	T4N3	1	1	0	0	0	4	0
7	13	T4N3	0	1	0	1	1	4	0
8	16	T4N3	1	1	3	0	1	4	0
9	16	T4N3	0	1	1	1	4	4	0
10	16	T3N1	2	0	0	2	3	4	0
11	16	T4N2	0	1	1	0	0	4	0
12	16	T3N2	0	1	0	0	3	4	0
13	16	T4N3	1	1	0	0	1	4	0

^a^All patients are M0

All patients completed treatment as planned, and no patient was lost to follow-up. All deaths were due to local recurrence or distant metastasis, and no patient died of other causes.

### Acute toxicity

All patients completed the planned treatment.

High grade acute organ toxicity concerning skin or esophagus occurred infrequently with addition of fludarabine at increasing doses to radiation (Table 1). Grade 1 radiation dermatitis was observed in 6 patients. Only one patient had a grade 2 skin toxicity in the supraclavicular fossa. Complete healing of skin lesions was observed within 1–2 weeks after completion of treatment. The severity of skin toxicity did not seem to increase with the dose of fludarabine.

Grade 1 esophagitis occurred in 11 patients, whereas only one grade 2 esophagitis could be observed. All symptoms recovered after radiochemotherapy. Lung toxicity was stated with grade 1 symptoms in 5 patients, and only one patient was observed with grade 3 pneumonitis according to RTOG/EORTC criteria (Table 1).

With regard to hematological complications, no thrombocytopenia was observed and anemia remained mild with 2 patients experiencing a grade 2 toxicity (Table 2). In all these patients, the nadir of hemoglobin occurred after the completion of radiotherapy. No transfusions were required. Grade 2 anemia occurred with a daily fludarabine dose of 16 mg/m^2^.

Neutropenia progressively increased with the dose of fludarabine (Table 1). At a daily dose of 16 mg/m^2^, 50 % of patients (three out of six) developed a grade 3 or 4 neutropenia. One of these latter two patients experienced fever above 40.0°C, but no infection was documented. This patient was hospitalized and received i.v. antibiotics for 8 days.

In all patients, grade 3 and 4 neutropenia occurred within the first 2 weeks after the completion of treatment and quickly recovered. However, all but one patient experienced a profound depletion of the lymphocyte count. Lymphocytopenia was found to increase with the dose of fludarabine. All lymphocyte subtypes were found to be depleted. The lymphocyte count progressively recovered but was still under pretreatment values at 3 months after treatment. No patient developed an opportunistic infection during the follow-up period.

One patient experienced acute grade 3 bronchopulmonary toxicity. A hospitalization of 2 weeks, with treatment with cortisone was required. In this patient, dyspnea rapidly resolved after the start of steroids without permanent impairment of pulmonary function.

According to the toxicity data, dose level of 13 mg/m^2^ was set as the MTD.

### CD4/CD8 count during and after the treatment

**Fig. 1:**
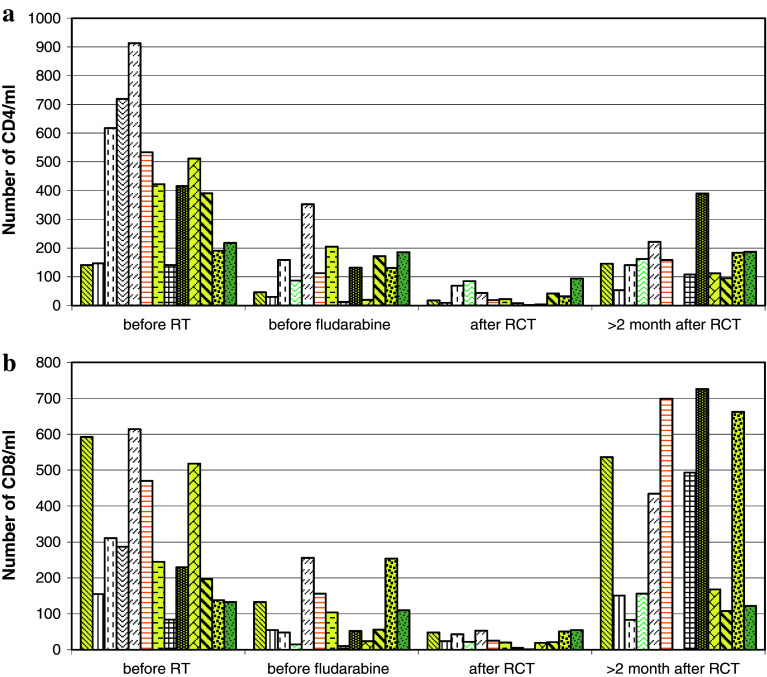
**a** Variation of the number of CD4 lymphocytes before, during and after treatment. **b** Variation of the number of CD8 lymphocytes before, during and after treatment

Lymphocytopenia (CD4 and CD8) was consistently observed in subjects who received a daily Fludarabine dose of 10 mg/m^2^ and higher. We observed a lymphocytopenia during the radiotherapy, and the depletion increased with the given dose of fludarabine. All patients recovered within 2 months after the end of the treatment. The details of the lymphocyte count during the treatment are reported in the Fig. 1a (CD4) and 1b (CD8).

### Survival

**Fig. 2:**
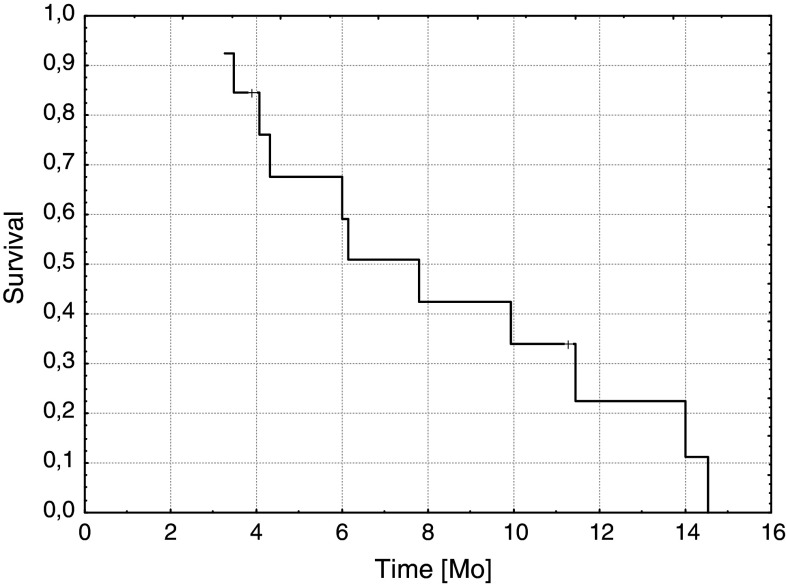
Overall survival. 1 overall survival rate was 22 %

Overall survival for the whole patient population assessed by the Kaplan–Meier analysis is given in Fig. 2. The 1-year survival rate was 22 %. The median follow-up was 6.5 months.

## Discussion

We initiated a phase I study with a normofractionated RT, and the main objective of this study was to establish the MTD for a regimen combining daily doses of fludarabine during the last 10 fractions of a standard radiotherapy course for patients with inoperable NSCLC. Fludarabine was chosen, while it has little toxicity on the mucosa and an estimated similar effect on the disease as gemcitabin, an highly effective nucleoside analogon. According to the definition used in our study, the MTD was reached for a daily fludarabine dose of 13 mg/m^2^. At the dose level 16 mg/m^2^, one out of six patients experienced a grade 4 neutropenia, and one of the six patients developed a grad 3 pneumonitis which recovered in 4 weeks. Apart from lymphocytopenia and neutropenia, no substantial other hematological toxicity was observed. Interestingly, we did not observe any opportunistic infections, although fludarabine specifically depletes lymphocytes (as shown in our study) and induces profound and prolonged immunosuppression. In part, this effect is mediated by sustained loss of STAT1 (signal transducer and activator of transcription 1), a molecule that is essential for cell-mediated immunity and STAT1-dependent gene transcription in lymphocytes (Cheson 1995; Frank et al. 1999). We did not observe significant esophageal or cutaneous toxicity. The rate of lung and esophageal toxicity observed in our study was inferior to the one reported in another recent phase I study comparing gemcitabin and radiotherapy in locally advanced non-small cell lung cancer (van Putten et al. 2003). Gemcitabin was administered weekly at a dose of 300 mg/m^2^. The authors described 3 patients with grade 3 toxicity, one pneumonitis, one upper gastrointestinal toxicity, and one esophagitis. In contrast to our study, gemcitabine and radiation did not result in hematological toxicities, except for temporary lymphopenia in 89 % of patients attributed to the radiotherapy.

This current study is the only one to investigate the combination of fludarabine with thoracic radiotherapy. A Belgian group explored concurrent fludarabine and radiotherapy in patients with intermediate to locally advanced head and neck squamous cell carcinomas (Gregoire et al. 2002). They reported an MTD of 17.5 mg/m^2^ with fludarabine and concomitant irradiation. The addition of fludarabine at increasing doses to radiation did not result in increased intensity or duration of skin or mucosal radiotoxicity compared to what was expected for radiation alone. At a daily dose of 17.5 mg/m^2^, two out of five patients developed grade 4 neutropenia. As in our study, all patients developed a fludarabine dose-dependent lymphocytopenia.

The exact mechanism of radiosensitization induced by fludarabine is unknown. Induction of double-strand breaks in DNA is considered to be one of the most important cytotoxic effects of radiotherapy. Several possible mechanisms for the radiosensitizing effects of gemcitabine have been discovered, including changes in nucleotide pools and cell cycle distribution. In vitro, fludarabine seems to impair homologous recombination, which suggests that radiation-induced DNA damage cannot be properly repaired and results in increased tumor cell killing. From preclinical studies, it is well known that fludarabine inhibits DNA rejoining, which is one possible mechanism of radiosensitization (Huang et al. 1990). Another report indicated that, after 24 h of fludarabine application, the drug induced an elimination of cells in the relatively radioresistant S phase of the cell cycle and also arrested the other cells in the G2/M phase, in which the cells are most radiosensitive (Gregoire et al. 1994c). The mechanisms by which fludarabine increases the radiation-induced reduction in cell survival in vitro are not yet fully understood. In an SA-NH mouse sarcoma cell line, fludarabine given 1 h prior to irradiation did not modify the rejoining of radiation-induced DNA DSB, measured by means of pulse-field gel electrophoresis (Gregoire and Hittelmann 1997). In contrast, it has been reported that fludarabine induced a complete inhibition of chromosome break repair in human lymphocytes after incubation for 30 min before irradiation (Jayanth and Hittelman 1991). Other investigations, however, have demonstrated that the repair of chemotherapy-induced DNA lesions is inhibited by fludarabine (Li et al. 1997).

In conclusion, in this study, the MTD of fludarabine when given daily for the last 10 fractions of a conventional fractionated radiotherapy regimen was determined to be 13 mg/m^2^ per day. We observed an additional toxicity with neutropenia, lymphocytopenia, and pneumonitis. With the exception of contradictory data on cisplatin and carboplatin, the role of radiosensitizers in NSCLC has not been evaluated in randomized studies. Because fludarabine is one of the strongest radiosensitizers known in NSCLC, the clinical usefulness of this combined approach should be further evaluated.
